# Evaluation of a new equation for estimating low-density lipoprotein cholesterol through the comparison with various recommended methods

**DOI:** 10.11613/BM.2021.010701

**Published:** 2020-12-15

**Authors:** Eduardo Martínez-Morillo, María García-García, María Angeles Luengo Concha, Luis Rello Varas

**Affiliations:** 1 Department of Clinical Biochemistry, Hospital Universitario Miguel Servet, Zaragoza, Spain; 2 Department of Clinical Biochemistry, Hospital del Oriente de Asturias, Arriondas, Asturias, Spain

**Keywords:** cholesterol, equation, evaluation, low-density lipoprotein, triglycerides

## Abstract

**Introduction:**

The accurate estimation of low-density lipoprotein cholesterol (LDL) is crucial for management of patients at risk of cardiovascular events due to dyslipidemia. The LDL is typically calculated using the Friedewald equation and/or direct homogeneous assays. However, both methods have their own limitations, so other equations have been proposed, including a new equation developed by Sampson. The aim of this study was to evaluate Sampson equation by comparing with the Friedewald and Martin-Hopkins equations, and with a direct LDL method.

**Materials and methods:**

Results of standard lipid profile (total cholesterol (CHOL), high-density lipoprotein cholesterol (HDL) and triglycerides (TG)) were obtained from two anonymized data sets collected at two laboratories, using assays from different manufacturers (Beckman Coulter and Roche Diagnostics). The second data set also included LDL results from a direct assay (Roche Diagnostics). Passing-Bablok and Bland-Altman analysis for method comparison was performed.

**Results:**

A total of 64,345 and 37,783 results for CHOL, HDL and TG were used, including 3116 results from the direct LDL assay. The Sampson and Friedewald equations provided similar LDL results (difference ≤ 0.06 mmol/L, on average) at TG ≤ 2.0 mmol/L. At TG between 2.0 and 4.5 mmol/L, the Sampson-calculated LDL showed a constant bias (- 0.18 mmol/L) when compared with the Martin-Hopkins equation. Similarly, at TG between 4.5 and 9.0 mmol/L, the Sampson equation showed a negative bias when compared with the direct assay, which was proportional (- 16%) to the LDL concentration.

**Conclusions:**

The Sampson equation may represent a cost-efficient alternative for calculating LDL in clinical laboratories.

## Introduction

The accurate estimation of low-density lipoprotein cholesterol (LDL) blood concentration is very important because large-scale evidence from randomised trials shows that statin therapy reduces the risk of atherosclerotic cardiovascular disease (ASCVD) proportionally to the absolute achieved reduction in LDL concentration (*1*, *2*). Consequently, LDL examination is recommended as the primary lipid analysis method for screening, diagnosis, and management of patients at risk of ASCVD due to dyslipidemia. The 2019 European Society of Cardiology/European Atherosclerosis Society (ESC/EAS) Guidelines for the management of dyslipidemias recommend treating patients to risk-stratified LDL target concentration: < 1.4 mmol/L, < 1.8 mmol/L, < 2.6 mmol/L, and < 3.0 mmol/L, for patients at very-high, high, moderate, and low ASCVD risk, respectively, with the 10-year risk of fatal ASCVD being estimated using the Systematic Coronary Risk Evaluation (SCORE) (*3*).

The reference method for LDL determination is the combination of ultracentrifugation and heparin-Mn^2+^ precipitation, named generically β-quantification (*4*). However, most clinical laboratories do not use this assay because it is an expensive, laborious, tough and time-consuming method, that also requires large sample volumes (*5*). Conversely, LDL is typically calculated using the Friedewald equation, which for the general population is fairly accurate and shows a very strong correlation with homogeneous assays for direct LDL determination (*6*, *7*). However, calculated LDL underestimates the actual LDL concentration in patients with high triglycerides (TG) concentration (> 2.0 mmol/L), especially if they show low LDL concentrations (*8*, *9*). For this reason, many laboratories provide a direct analysis with homogeneous LDL assays at high TG concentrations, but although direct methods are considered a proper alternative to β-quantification, they have their own limitations in terms of reliability and specificity, specially in patients with atypical lipoproteins (*10*).

To overcome the limitations of the Friedewald equation, new equations have been proposed over the years (*11*-*13*). Particularly, the Martin-Hopkins equation has shown to provide a more accurate estimation of LDL measured by direct assay, especially in individuals with LDL < 1.8 mmol/L and in samples with TG concentration up to 4.5 mmol/L (*14*). However, although the Martin-Hopkins equation provides a more accurate guideline risk classification than the Friedewald equation, it does not correlate with LDL measured directly by β-quatification in samples with high TG concentration (> 4.5 mmol/L) (*15*-*17*).

An expert panel from the EAS and the European Federation of Clinical Chemistry and Laboratory Medicine (EFLM) have recently published a set of recommendations on the quantification of atherogenic lipoproteins, including various key recommendations referred to the test measurement: (a) follow-up of measured or calculated LDL of a patient, from baseline to on-treatment measurements, should be ideally performed with the same method; (b) the Martin-Hopkins equation may be preferable to the Friedewald equation for calculation of LDL in patients with low LDL concentration (< 1.8 mmol/L) and/or TG concentration between 2.0 and 4.5 mmol/L; (c) direct LDL assays should be used for assessment of LDL when TG concentration is higher than 4.5 mmol/L (*8*, *17*, *18*).

Very recently, a new equation to estimate LDL, based on β-quantification, have been proposed by Sampson *et al*. According to their results, this equation provides a more accurate calculation of LDL than the Friedewald and Martin-Hopkins equations in patients with low LDL concentration and/or hypertriglyceridemia (TG ≤ 9.0 mmol/L) (*19*).

Thus, the hypothesis of this study was that this novel equation (the Sampson equation), developed with LDL results obtained by the reference method, may replace the previously mentioned equations and direct assays for LDL estimation. Therefore, the aim was to compare Sampson-calculated LDL concentrations with the results derived from the Friedewald and Martin-Hopkins equations, and with the LDL concentrations measured with a direct method.

## Materials and methods

### Study design

This was a restrospective study where results of standard lipid profile were obtained from two data sets collected at the biochemistry laboratories of: (i) a tertiary-level hospital (Hospital Universitario Miguel Servet (HUMS)) from Zaragoza (Spain), by enzymatic colorimetric assays using the AU5800 (Beckman Coulter Inc, Brea, USA), with data exported from the laboratory information system (LIS) Modulab (Werfen, Barcelona, Spain); (ii) a secondary-level hospital (Hospital del Oriente de Asturias (HOA)) from Asturias (Spain), by enzymatic colorimetric assays with the Cobas c501 (Roche Diagnostics GmbH, Mannheim, Germany), with data exported from the LIS Omega 3000, Roche Diagnostics. The goal of this design was to verify whether the differences observed between the evaluated methods for estimating LDL concentration were consistent, regardless of the laboratory size, the type of hospital and the manufacturer’s test kits. Patient data were extracted and anonymized. Therefore, informed consent and ethical approval were not required.

Results of total cholesterol (CHOL), high-density lipoprotein cholesterol (HDL) and TG from serum samples assayed routinely at HUMS and HOA were collected. The exclusion criteria were: samples without results for any of the three parameters and samples with TG concentration above 9.0 mmol/L. The data set from HOA also included LDL results obtained with a direct assay (LDLC3, Roche Diagnostics), in serum samples with TG concentration ≥ 3.4 mmol/L, according to an internal protocol, since calculated LDL was considered less acurate in these samples. Direct LDL analysis was not performed at HUMS, so data were not available. Data from HUMS were collected between January 2019 and February 2020, while data collection from HOA was extended from January 2015 to December 2019, in order to obtain a similar number of results from both laboratories.

Although multiple comparisons of LDL results were performed, the Sampson equation was evaluated by comparing the estimated LDL concentrations with those provided by the consensus-based recommended method from EAS/EFLM: (i) the Friedewald equation at TG ≤ 2.0 mmol/L, (ii) the Martin-Hopkins equation at TG between 2.0-4.5 mmol/L, (iii) and the direct assay at TG between 4.5-9.0 mmol/L.

### Methods

The equations used to calculate the LDL concentration were:

Friedewald equation (in mg/dL):


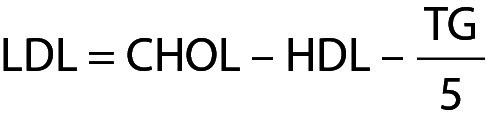


Friedewald equation (in mmol/L):


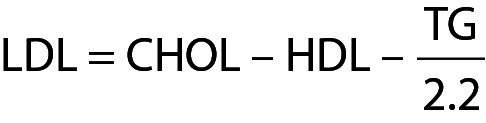


Martin-Hopkins equation:


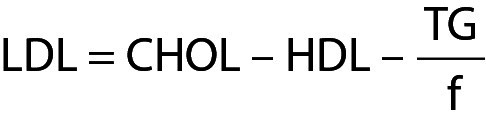


Where f - adjustable value (factor) ranging from 3.1 to 11.9 (results in mg/dL), obtained from the 180-cell factor table (*14*). For conversion to the International System of Units (SI), multiply f by 0.43658 (results in mmol/L).

Sampson equation (in mg/dL):


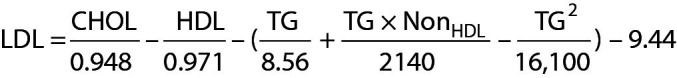


Sampson equation (in mmol/L):


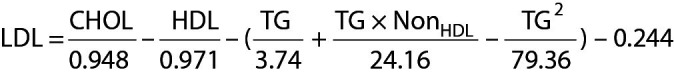


where:

LDL - low-density lipoprotein cholesterol, CHOL - total cholesterol, HDL - high-density lipoprotein cholesterol, TG - triglycerides and Non_HDL_ represents the difference of CHOL – HDL.

### Statistical analysis

Data are presented as numbers and percentages, medians with interquartile ranges (IQR), or mean of differences and mean absolute difference (MAD) with standard deviations. Calculation of LDL concentration with the three equations was performed by using the software Microsoft Excel for Office 365 MSO, version 16.0 (Microsoft Corporation, Redmond, USA). Likewise, data were also analysed and plotted with Microsoft Excel. Passing-Bablok regression and Bland-Altman plot analysis for method comparisons were performed with MedCalc, version 18.11.3 (MedCalc Software Ltd, Ostend, Belgium). The normal distribution was evaluated by the Kolmogorov-Smirnov test. The Wilcoxon signed-rank test and the Mann-Whitney U-test were performed for comparison between paired and independent samples, respectively.

Association between variables was assessed by the Pearson’s or Spearman’s rank correlation coefficients. A 2-sided P value ≤ 0.05 was considered statistically significant.

## Results

Data from HUMS and HOA included 64,524 and 37,802 results for CHOL, HDL and TG, respectively. Moreover, a total of 3116 LDL results from the direct assay were used. After initial analysis, serum samples with CHOL concentration above 10.4 mmol/L were excluded, showing an inverse trend between the Sampson- and Friedewald-calculated LDL results, and analysed separately (data not shown). Therefore, 64,345 and 37,783 results remained for further analysis (Table 1).

**Table 1:** Results obtained from two laboratories by using assays from different manufacturers

	**Distribution of results (N)** **based on TG** **concentration**			**Lipid concentration** **in mmol/L**		
	**≤ 2.0** **mmol/L**	**2.0-4.5 mmol/L**	**4.5-9.0 mmol/L**	**CHOL median (IQR)**	**HDL median (IQR)**	**TG median (IQR)**
HUMS	42,795	18,482	3068	5.25 (4.45-6.05)	1.34 (1.14-1.60)	1.39 (0.94-2.24)
HOA	29,185	7493	1105	5.04 (4.24-5.82)	1.37 (1.11-1.66)	1.31 (0.95-1.90)
Data collected from serum samples with TG concentration up to 9.0 mmol/L. Results from samples with CHOL concentration above 10.4 mmol/L were removed. HUMS - Hospital Universitario Miguel Servet. HOA - Hospital del Oriente de Asturias. IQR - Interquartile range. TG – triglycerides. CHOL – total cholesterol. HDL - high-density lipoprotein cholesterol.						

**Figure 1:**
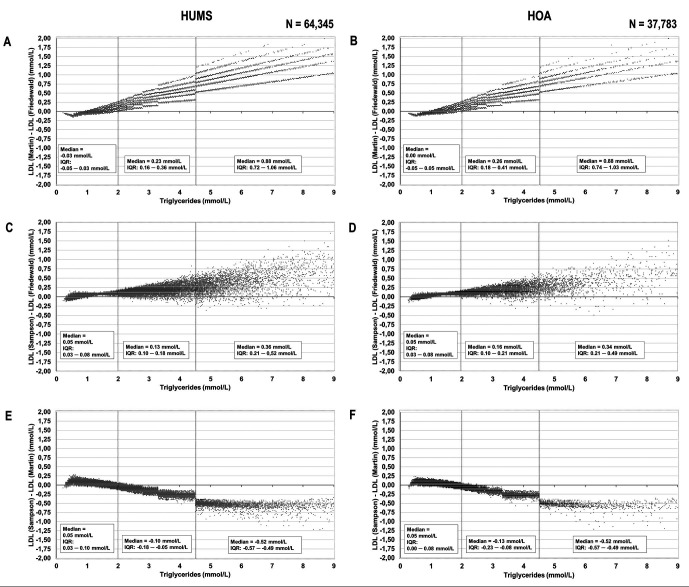
Comparison of LDL concentration obtained by using the Sampson, Martin-Hopkins and Friedewald equations with data from HUMS (A, C and E) and HOA (B, D and F). The median and interquartile range (IQR) of LDL differences are shown below. LDL - low-density lipoprotein cholesterol. HUMS - Hospital Universitario Miguel Servet. HOA - Hospital del Oriente de Asturias. TG – triglycerides. CHOL – total cholesterol. HDL - high-density lipoprotein cholesterol.

First, a comparison between the LDL concentration provided by the Sampson, Friedewald, and Martin-Hopkins equations was performed (Figure 1). Thus, differences in LDL concentration were plotted against the TG concentration range (0-9.0 mmol/L). Figure 1 shows as the differences observed between the calculated LDL, although statistically significant (P < 0.001) due to the large sample size, were very similar when data from HUMS and HOA were used. Thus, for instance, the medians of LDL differences between the Sampson and Friedewald equations were: 0.05 *vs* 0.05 mmol/L, at TG ≤ 2.0 mmol/L; 0.13 *vs* 0.16 mmol/L, at TG between 2.0-4.5 mmol/L; and 0.36 *vs* 0.34 mmol/L, at TG between 4.5-9.0 mmol/L (Figures 1C and 1D).

Additionally, obtained LDL results with the three equations were very similar at TG concentration ≤ 2.0 mmol/L (medians of LDL differences between 0 and 0.05 mmol/L), with differences ≤ 0.25 mmol/L in most samples. However, at TG concentration above 2.0 mmol/L, the results provided by the Martin-Hopkins and Sampson equations were mostly higher than those obtained with the Friedewald equation (medians of LDL differences ranging from 0.23 to 0.88 mmol/L, and from 0.13 to 0.36 mmol/L, respectively), increasing these differences gradually with TG concentration. Figure 1 also shows as the Sampson equation provided lower LDL concentrations than the Martin-Hopkins equation at TG > 2.0 mmol/L. This difference increased to values of - 0.25 mmol/L, on average, at TG between 2.0-4.5 mmol/L, and was mainly constant and with a value of - 0.50 mmol/L, on average, at TG between 4.5-9.0 mmol/L.

**Figure 2:**
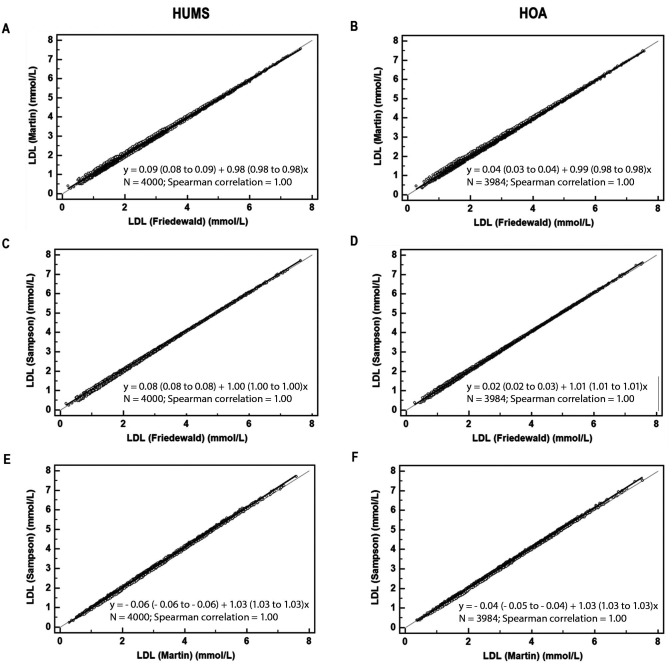
Passing-Bablok regression of LDL obtained with the Sampson, Martin-Hopkins and Friedewald equations, using data from HUMS (A, C and E) and HOA (B, D and F), with triglycerides concentration up to 2.0 mmol/L. The 95% confidence intervals for intercept and slope are shown within parentheses. Samples were selected based on the following eight LDL intervals with similar length: ≤ 1.4, 1.4-1.8, 1.8-2.6, 2.6-3.0, 3.0-3.4, 3.4-4.1, 4.1-4.9, and ≥ 4.9 mmol/L. Five-hundred samples, when available, were randomly selected from each group. LDL - low-density lipoprotein cholesterol. HUMS - Hospital Universitario Miguel Servet. HOA - Hospital del Oriente de Asturias.

**Figure 3:**
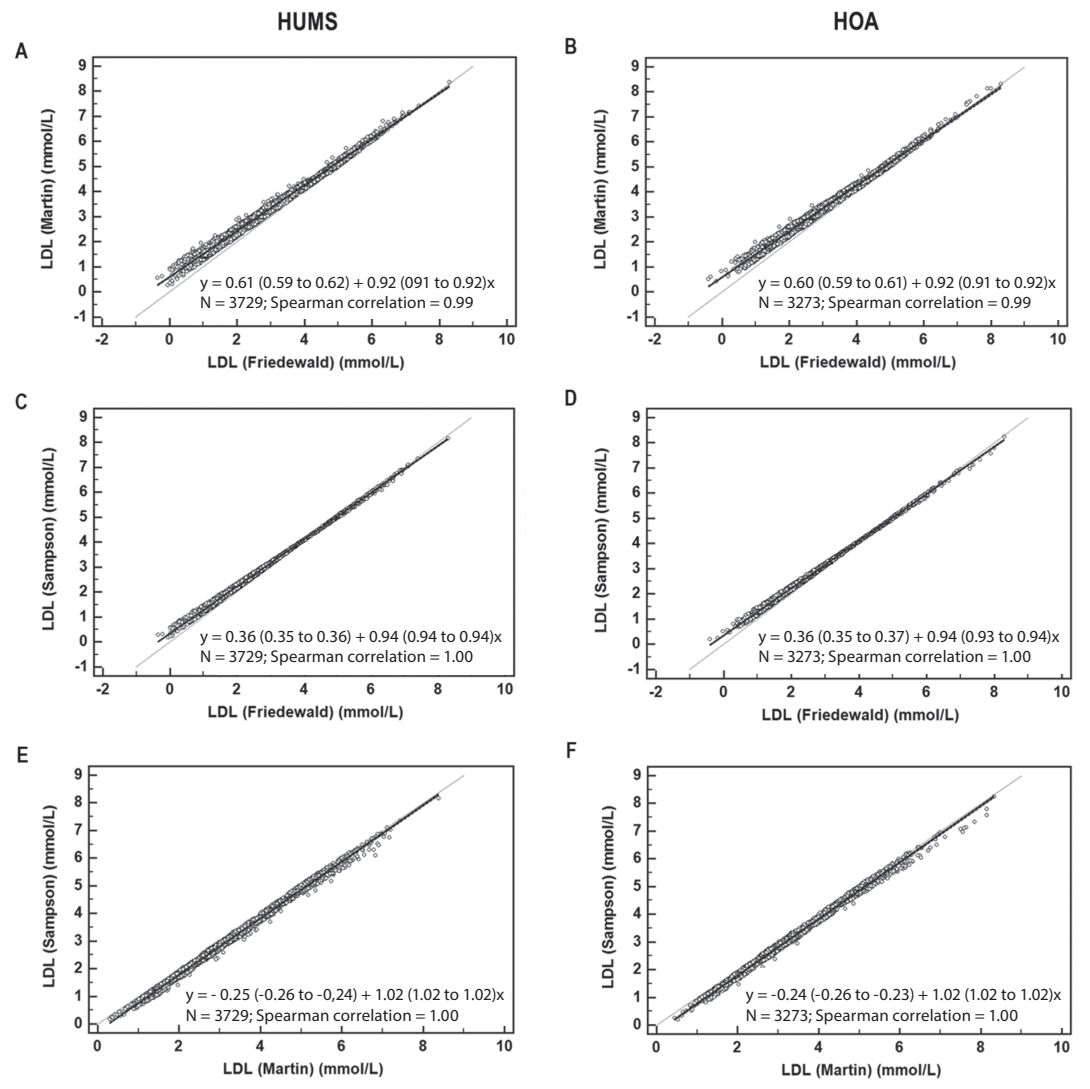
Passing-Bablok regression of LDL obtained with the Sampson, Martin-Hopkins and Friedewald equations, using data from HUMS (A, C and E) and HOA (B, D and F), with triglycerides concentration between 2.0 and 4.5 mmol/L. The 95% confidence intervals for intercept and slope are shown within parentheses. Samples were selected based on the following eight LDL intervals with similar length: ≤ 1.4, 1.4-1.8, 1.8-2.6, 2.6-3.0, 3.0-3.4, 3.4-4.1, 4.1-4.9, and ≥ 4.9 mmol/L. Five-hundred samples, when available, were randomly selected from each group. LDL - low-density lipoprotein cholesterol. HUMS - Hospital Universitario Miguel Servet. HOA - Hospital del Oriente de Asturias.

When comparing the calculated LDL at TG ≤ 2.0 mmol/L, the results were very similar throughout the entire LDL concentration interval (Figure 2). The Bland-Altman plot analysis showed constant and proportional differences lower or equal to 0.06 mmol/L (95% confidence interval (CI): - 0.13 to 0.17) and 1.7% (95%CI: - 7.9 to 10.2), on average, respectively. At TG between 2.0-4.5 mmol/L, the comparison of the LDL-calculated concentration showed that the Martin-Hopkins and Sampson equations estimated mostly higher LDL results than the Friedewald equation, specially at concentrations < 4 mmol/L (Figure 3). Difference in calculated LDL between the Martin-Hopkins and Sampson equations was mostly constant, with an average difference of - 0.18 mmol/L (95%CI: - 0.39 to 0.03) with the Sampson equation, according to the Bland-Altman analysis. Differences among equations at TG between 4.5-9.0 mmol/L were even higher, with the Sampson-calculated LDL showing an constant difference of - 0.54 mmol/L, on average, when compared with the LDL estimated with the Martin-Hopkins equation (data not shown).

**Figure 4:**
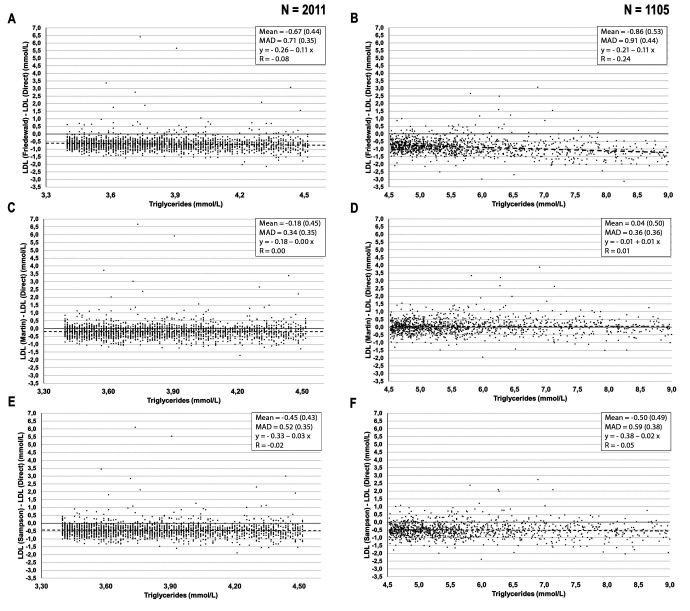
Comparison of LDL concentration obtained by using the Sampson, Martin-Hopkins and Friedewald equations against the results from the direct assay, using data from HOA and with triglycerides concentration between 3.4-4.5 mmol/L (A, C and E) and 4.5-9.0 mmol/L (B, D and F). The mean of differences and mean absolute difference (MAD) with the standard deviations within parentheses, the regression line (dashed line) and equation, and the correlation coefficient (R) are shown below. LDL - low-density lipoprotein cholesterol. HOA - Hospital del Oriente de Asturias.

**Figure 5:**
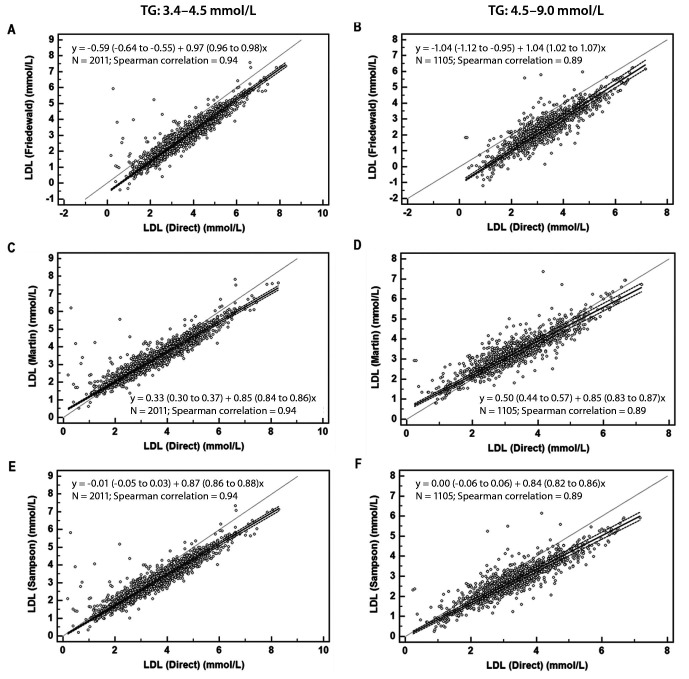
Passing-Bablok regression of LDL obtained with the Sampson, Martin-Hopkins and Friedewald equations versus the LDL measured with the direct assay, using data from HOA and with TG concentration between 3.4-4.5 mmol/L (A, C and E) and 4.5-9.0 mmol/L (B, D and F). The 95% confidence intervals for intercept and slope are shown within parentheses. The dashed lines represent the 95% confidence interval for the regression line. LDL - low-density lipoprotein cholesterol. HOA - Hospital del Oriente de Asturias.

On the other hand, a comparison between the calculated LDL concentrations and the direct LDL measurement, at TG between 3.4-4.5 mmol/L and 4.5-9.0 mmol/L, respectively, was performed (Figures 4 and 5). The aim was to compare the performance of these equations at TG concentrations below and above 4.5 mmol/L. Figure 4 shows that the Friedewald equation calculated mostly lower LDL concentrations than those obtained with the direct assay, with differences growing from - 0.6 mmol/L to - 1.3 mmol/L, on average, as TG concentration increased. The Martin-Hopkins equation estimated more similar LDL concentrations than the other two equations (MAD: 0.34 and 0.36 (Martin-Hopkins) *vs* 0.71 and 0.91 (Friedewald) and *vs* 0.52 and 0.59 (Sampson), respectively), with an average difference of aproximately - 0.2 and 0 mmol/L, at TG between 3.4-4.5 and 4.5-9.0 mmol/L, respectively. Moreover, the Sampson-calculated LDL concentrations were mostly lower than directly measured LDL results, with a constant difference of - 0.5 mmol/L, on average, throughout the entire TG concentration interval.

Figure 5 shows as the calculated LDL results were different to those obtained with the direct assay, depending on the actual LDL concentration. Thus, the Friedewald-calculated LDL was mostly lower, with negative and constant differences of - 0.7 mmol/L (95%CI: - 1.5 to 0.2) and - 0.9 mmol/L (95%CI: - 1.9 to 0.2), on average, at TG between 3.4-4.5 mmol/L and 4.5-9.0 mmol/L, respectively. When the Martin-Hopkins-calculated LDL and the directly measured LDL were compared, constant and proportional differences were observed, with higher Martin-Hopkins-calculated results at low LDL concentrations and lower calculated results at high LDL concentrations, specially at TG between 4.5-9.0 mmol/L. Moreover, the Sampson equation estimated mostly lower LDL concentrations than those measured with the direct assay, being these differences proportional to the LDL concentration, with average percentages of - 13% (95%CI: - 43 to 17) and - 16% (95%CI: - 53 to 20).

## Discussion

To our knowledge, this is the first study that evaluates the Sampson equation using real data (> 100,000 results from the standard lipid profile) from two different laboratories and with assays from different manufacturers.

The results obtained showed that the Sampson and Friedewald equations estimated very similar LDL results at TG ≤ 2.0 mmol/L, regardless of the LDL concentration. At higher TG concentration, the Sampson-calculated LDL was higher, not calculating negative results as it occurs with the Friedewald equation when the actual LDL concentration is very low (data not shown). Therefore, this novel equation does not seem to underestimate the LDL concentration or, at least, the negative bias is much lower than that by the Friedewald equation. However, the Sampson equation estimated lower LDL concentration at high CHOL concentrations, but in all these patients the LDL concentration was elevated, when an accurate estimation is less critical to treatment goals.

At TG between 2.0-4.5 mmol/L, the Sampson-calculated LDL showed a small negative bias when compared with the Martin-Hopkins equation, regardless of the LDL concentration. Similarly, at TG between 4.5-9.0 mmol/L, the Sampson equation showed a negative bias when compared with the direct assay, which was proportional to the LDL concentration. However, it is necessary to take into account that although the Martin-Hopkins-calculated LDL was more similar to that provided by the direct method, it appeared to be overestimated at low LDL concentrations and underestimated at high concentrations. Moreover, although it has been demonstrated that the Martin-Hopkins equation performs better than the Friedewald equation at TG concentrations between 2.0-4.5 mmol/L, and when the LDL concentration is low, Palmer *et al.* found that at TG concentration above 4.5 mmol/L this equation overestimates the LDL concentration obtained with β-quantification, especially when the LDL is below 4 mmol/L, and may understimate the actual LDL concentration at very high concentrations (*14*-*16*). On the other hand, direct homogenous LDL assays perform better than the Friedewald equation at high TG concentrations and generally meet the National Cholesterol Education Program (NCEP) requirements (*10*). However, these methods have progressively poor performance as the TG concentration of the specimens increases, showing a trend from negative to positive values (*20*, *21*). Thus, discrepancies between homogeneous assays and β-quantification are found mostly in hypertriglyceridemic subjects, with some studies showing positive but minor biases (< 5%, on average), with no consistent pattern for the frequency of discordant LDL results with triglyceride concentration (*22*-*24*). Therefore, the negative bias observed in this study with the Sampson equation when compared with the Martin-Hopkins-calculated LDL and with the directly measured LDL concentrations may not be extensible to the reference method or it is probably less. Sampson *et al.* also found that the Martin-Hopkins equation, which is based on the Vertical Auto Profile test (an ultracentrifugation density-based separation procedure different to the reference method), misclassified 31.9% of patients with TG concentration between 4.5-9.0 mmol/L into some treatment categories, mainly falsely increasing the LDL result. However, the Sampson equation had a total error of 22.3%, with patients been misclassified in both ways, by falsely increasing or decreasing the LDL result (*19*).

In conclusion, this study shows that the Sampson equation can be implemented in clinical laboratories, providing an acceptable performance. This equation has several advantages: (i) It can replace the Friedewald equation, since at low TG concentrations (≤ 2.0 mmol/L), when Friedewald equation is trustable, both equations calculate very similar LDL concentrations; (ii) Although this equation is more complicated than other previously published, it can be automatically calculated by most LIS, unlike the Martin-Hopkins equation; (iii) It can be used in patients with TG concentration up to 9.0 mmol/L, saving costs and complying the EAS/EFLM recommendation to use the same method for on-treatment follow-up, to attenuate errors in treatment decisions due to marked between-methods variations (*7*, *25*, *26*). The main disadvantages are: (i) The Sampson-calculated LDL depends upon three laboratory assays (CHOL, HDL, and TG), meaning that three measurement errors are involved which inevitably introduce calculation variability. However, we have obtained very similar results using data from two laboratories obtained with assays from different manufacturers; (ii) The equation appears to have a negative proportional bias respect to the direct assay.

The main limitation of this study was not having LDL results obtained by the reference method. However, the obtained results will allow clinical laboratories to estimate which differences could be found if they decide to implement this new equation, based on β-quantification, into their daily routine.

## References

[1] Collins R Reith C Emberson J Armitage J Baigent C Blackwell L Interpretation of the evidence for the efficacy and safety of statin therapy. Lancet. 2016;388:2532–61. 10.1016/S0140-6736(16)31357-5 27616593

[2] Sabatine MS Giugliano RP Keech AC Honarpour N Wiviott SD Murphy SA Evolocumab and Clinical Outcomes in Patients with Cardiovascular Disease. N Engl J Med. 2017;376:1713–22. 10.1056/NEJMoa1615664 28304224

[3] Authors/Task Force Members, ESC Committee for Practice Guidelines (CPG), ESC National Cardiac Societies 2019 ESC/EAS guidelines for the management of dyslipidaemias: Lipid modification to reduce cardiovascular risk. Atherosclerosis. 2019;290:140–205. 10.1016/j.atherosclerosis.2019.08.014 31591002

[4] Bachorik PS Ross JW National Cholesterol Education Program recommendations for measurement of low-density lipoprotein cholesterol: executive summary. The National Cholesterol Education Program Working Group on Lipoprotein Measurement. Clin Chem. 1995;41:1414–20. 10.1093/clinchem/41.10.1414 7586510

[5] Rifai N, Warnick GR, Dominiczak MH, editors. Handbook of Lipoprotein Testing. 2nd ed. Washington, DC: AACC Press; 2000.

[6] Razi F Forouzanfar K Bandarian F Nasli-Esfahani E LDL-cholesterol measurement in diabetic type 2 patients: a comparison between direct assay and popular equations. J Diabetes Metab Disord. 2017;16:43. 10.1186/s40200-017-0326-2 29142884 PMC5670525

[7] Sung K-C Kwon CH Lee MY Kwon M-J Lee JH Jung M-H Comparison of Low-Density Lipoprotein Cholesterol Concentrations by Direct Measurement and by Friedewald Calculation. Am J Cardiol. 2020;125:866–73. 10.1016/j.amjcard.2019.12.036 31928718

[8] Langlois MR Nordestgaard BG Langsted A Chapman MJ Aakre KM Baum H Quantifying atherogenic lipoproteins for lipid-lowering strategies: consensus-based recommendations from EAS and EFLM. Clin Chem Lab Med. 2020;58:496–517. 10.1515/cclm-2019-1253 31855562

[9] Lee J Jang S Jeong H Ryu O-H Validation of the Friedewald formula for estimating low density lipoprotein cholesterol: the Korea National Health and Nutrition Examination Survey, 2009 to 2011. Korean J Intern Med. 2020;35:150–9. 10.3904/kjim.2017.233 29551052 PMC6960042

[10] Ramasamy I Update on the laboratory investigation of dyslipidemias. Clin Chim Acta. 2018;479:103–25. 10.1016/j.cca.2018.01.015 29336935

[11] Anandaraja S Narang R Godeswar R Laksmy R Talwar KK Low-density lipoprotein cholesterol estimation by a new formula in Indian population. Int J Cardiol. 2005;102:117–20. 10.1016/j.ijcard.2004.05.009 15939107

[12] Chen Y Zhang X Pan B Jin X Yao H Chen B A modified formula for calculating low-density lipoprotein cholesterol values. Lipids Health Dis. 2010;9:52. 10.1186/1476-511X-9-52 20487572 PMC2890624

[13] de Cordova CMM de Cordova MM A new accurate, simple formula for LDL-cholesterol estimation based on directly measured blood lipids from a large cohort. Ann Clin Biochem. 2013;50:13–9. 10.1258/acb.2012.011259 23108766

[14] Martin SS Blaha MJ Elshazly MB Toth PP Kwiterovich PO Blumenthal RS Comparison of a novel method vs the Friedewald equation for estimating low-density lipoprotein cholesterol levels from the standard lipid profile. JAMA. 2013;310:2061–8. 10.1001/jama.2013.280532 24240933 PMC4226221

[15] Martin SS Giugliano RP Murphy SA Wasserman SM Stein EA Ceška R Comparison of Low-Density Lipoprotein Cholesterol Assessment by Martin/Hopkins Estimation, Friedewald Estimation, and Preparative Ultracentrifugation: Insights From the FOURIER Trial. JAMA Cardiol. 2018;3:749–53. 10.1001/jamacardio.2018.1533 29898218 PMC6143070

[16] Palmer MK Barter PJ Lundman P Nicholls SJ Toth PP Karlson BW Comparing a novel equation for calculating low-density lipoprotein cholesterol with the Friedewald equation: A VOYAGER analysis. Clin Biochem. 2019;64:24–9. 10.1016/j.clinbiochem.2018.10.011 30365923

[17] Langlois MR Chapman MJ Cobbaert C Mora S Remaley AT Ros E Quantifying Atherogenic Lipoproteins: Current and Future Challenges in the Era of Personalized Medicine and Very Low Concentrations of LDL Cholesterol. A Consensus Statement from EAS and EFLM. Clin Chem. 2018;64:1006–33. 10.1373/clinchem.2018.287037 29760220

[18] Nordestgaard BG Langsted A Mora S Kolovou G Baum H Bruckert E Fasting is not routinely required for determination of a lipid profile: clinical and laboratory implications including flagging at desirable concentration cut-points-a joint consensus statement from the European Atherosclerosis Society and European Federation of Clinical Chemistry and Laboratory Medicine. Eur Heart J. 2016;37:1944–58. 10.1093/eurheartj/ehw152 27122601 PMC4929379

[19] Sampson M Ling C Sun Q Harb R Ashmaig M Warnick R A New Equation for Calculation of Low-Density Lipoprotein Cholesterol in Patients With Normolipidemia and/or Hypertriglyceridemia. JAMA Cardiol. 2020;5:540–8. 10.1001/jamacardio.2020.0013 32101259 PMC7240357

[20] Miller WG Waymack PP Anderson FP Ethridge SF Jayne EC Performance of four homogeneous direct methods for LDL-cholesterol. Clin Chem. 2002;48:489–98. 10.1093/clinchem/48.3.489 11861439

[21] Nauck M Warnick GR Rifai N Methods for measurement of LDL-cholesterol: a critical assessment of direct measurement by homogeneous assays versus calculation. Clin Chem. 2002;48:236–54. 10.1093/clinchem/48.2.236 11805004

[22] Esteban-Salan M Aguilar-Doreste JA Arranz-Pena ML Juve-Cuxart S Gich-Salarich I Zapico-Muniz E Multicentric evaluation of the homogeneous LDL-cholesterol Plus assay: comparison with beta-quantification and Friedewald formula. Clin Biochem. 2008;41:1402–9. 10.1016/j.clinbiochem.2008.07.014 18722364

[23] Miida T Nishimura K Okamura T Hirayama S Ohmura H Yoshida H A multicenter study on the precision and accuracy of homogeneous assays for LDL-cholesterol: comparison with a beta-quantification method using fresh serum obtained from non-diseased and diseased subjects. Atherosclerosis. 2012;225:208–15. 10.1016/j.atherosclerosis.2012.08.022 22980501

[24] Miller WG Myers GL Sakurabayashi I Bachmann LM Caudill SP Dziekonski A Seven direct methods for measuring HDL and LDL cholesterol compared with ultracentrifugation reference measurement procedures. Clin Chem. 2010;56:977–86. 10.1373/clinchem.2009.142810 20378768 PMC4687457

[25] Choi H Shim J-S Lee MH Yoon YM Choi DP Kim HC Comparison of Formulas for Calculating Low-density Lipoprotein Cholesterol in General Population and High-risk Patients with Cardiovascular Disease. Korean Circ J. 2016;46:688–98. 10.4070/kcj.2016.46.5.688 27721861 PMC5054182

[26] Karkhaneh A Bagherieh M Sadeghi S Kheirollahi A Evaluation of eight formulas for LDL-C estimation in Iranian subjects with different metabolic health statuses. Lipids Health Dis. 2019;18:231. 10.1186/s12944-019-1178-1 31883533 PMC6935216

